# Immediate and longer-term impacts of fetal surveillance education on workforce knowledge and cognitive skills

**DOI:** 10.12688/mep.19656.1

**Published:** 2023-07-19

**Authors:** Mark Beaves, Nathan Zoanetti, Euan M Wallace, Kirsten R Palmer

**Affiliations:** 1 Royal Australian and New Zealand College of Obstetricians and Gynaecologists, MELBOURNE, Victoria, 3004, Australia; 2 Dept of Obstetrics and Gynaecology, Monash University, Clayton, Victoria, 3800, Australia; 3 Research, Australian Council for Educational Research, Camberwell, Victoria, 3124, Australia; 4 Victorian Government, Department of Health, MELBOURNE, Victoria, 3000, Australia; 5 Obstetrics and Gynaecology, Monash Health, Clayton, Victoria, 3168, Australia

**Keywords:** fetal surveillance, medical education, midwifery education, CTG, assessment, medical education program, CTG education, fetal surveillance knowledge, fetal surveillance education, cardiotocograph

## Abstract

**Background:** Following the development of the Royal Australian College of Obstetricians and Gynaecologists Intrapartum Fetal Surveillance Guideline in 2003, an education program was developed to support guideline implementation and clinical practice. It was intended that improved clinician knowledge, particularly of cardiotocography, would reduce rates of intrapartum fetal morbidity and mortality. The program contains a multiple-choice assessment, designed to assess fetal surveillance knowledge and the application of that knowledge. We used the results of this assessment over time to evaluate the impact of the education program on clinicians’ fetal surveillance knowledge and interpretive skills, in the immediate and longer-term.

**Methods:** We undertook a retrospective analysis of the assessment results for all participants in the Fetal Surveillance Education Program, between 2004 and 2018. Classical Test Theory and Rasch Item Response Theory analysis were used to evaluate the statistical reliability and quality of the assessment, and the measurement invariance or stability of the assessments over time. Clinicians’ assessment scores were then reviewed by craft group and previous exposure to the program.

**Results:** The results from 64,430, broadly similar assessments, showed that participation in the education program was associated with an immediate improvement in clinician performance in the assessment. Performance improvement was sustained for up to 18 months following participation in the program and recurrent participation was associated with progressive improvements. These trends were observed for all craft groups (consultant obstetricians, doctors in training, general practitioners, midwives, student midwives).

**Conclusions:** These findings suggest that the Fetal Surveillance Education Program has improved clinician knowledge and the associated cognitive skills over time. The stable difficulty of the assessment tool means any improvement in clinician’s results, with ongoing exposure to the program, can be reliably assessed and demonstrated. Importantly this holds true for all craft groups involved in intrapartum care and the interpretation of cardiotocography.

## Introduction

Twenty years ago, in Australia and New Zealand no high-quality educational resources in fetal surveillance for clinicians existed ^1^. In response to local, national, and international calls for better training in intrapartum fetal surveillance ^1– 6^ the Royal Australian and New Zealand College of Obstetricians and Gynaecologists (RANZCOG) developed and introduced the Fetal Surveillance Education Program (FSEP) ^7^.

The FSEP is a full-day program that covers uteroplacental function, the physiology of fetal heart rate control, the normal and abnormal cardiotocograph (CTG), the clinical picture, maternal heart rate monitoring, uterine hyperstimulation, behavioural factors impacting CTG interpretation, case study workshops, and the 60-item multiple choice question (MCQ) assessment ^7^. Each participant receives a results letter and a Graphical Item Map ^8^ intended to feedback strengths and weaknesses in fetal surveillance, as determined by the assessment. The intention of FSEP, including the assessment tool, was to reduce intrapartum-related fetal morbidity and mortality. It was hoped that multidisciplinary education, based on the physiology and pathophysiology of fetal heart rate control, might improve clinical outcomes by improving CTG use, interpretation, and management ^3– 6^.

Here we evaluate clinician performance on the RANZCOG FSEP assessment over time, to determine whether FSEP confers improved knowledge and cognitive skills, across all craft groups, in CTG interpretation and management. 

## Methods

### FSEP assessment development

In the first three years of FSEP (2004–2006), participants completed two, near identical 20 item multi-choice question (MCQ) assessments, one immediately prior to, and one immediately following the education program. These initial MCQ based assessments, for which the construct validity has been previously described ^9^, consisted of questions exploring five knowledge domains: definitions, fetal physiology, the application of definitions, the application of physiology, and management and decision making. The 20 items were combined to provide a spectrum of question difficulty. 

In 2007, the pre-education assessment was discontinued. From 2007 to 2009 the post-education assessment was gradually increased to 60-items to increase the statistical reliability ^9^ of the assessment tool. Data from the period of evolvement from 20 to 60 MCQs (2007–2009) are not included here, due to ongoing changes in MCQ numbers and assessment reliability. Only data from the 20 question pre- and post-test (2004–2006) and the 60-item MCQ test (2010–2018) are included in this analysis. 

### Data extraction

All data were derived from the RANZCOG FSEP database, an electronic record of individual clinicians FSEP assessment performance between 2004 and 2018. Data on prior FSEP attendance is automatically reported from the database, enabling comparison between first time participants, and those with one, or more previous exposures to the education program and the assessment tool.

### Outcome measures

The variables extracted from the database included the average score, the average score for the first exposure to the assessment and the average score for those with repeated exposures per year per craft group over the analysis period. The craft groups for the program participants were identified as: obstetricians (O&G), registrars/senior obstetric trainees (Reg), residents/junior obstetric trainees (Res), general practitioners (GP), obstetric trained GPs (GP Obs), midwives (Mid) and student midwives (Stu Mid). Graduate/first year midwives (Grad Mid) were included as a separate cohort from 2012.

### Statistical analyses

We analysed the initial 20 item pre – and post-education assessments used between 2004 – 2006 using Classical Test Theory (CTT) and Item Response Theory (IRT), evaluating the performance of the individual questions (discrimination, distracter response frequencies, distracter point biserial and the percentage of correct responses). We also evaluated the overall assessment performance (Cronbach alpha ^10^, mean and standard deviation of population scores) and compared the two assessments.

To assess immediate impact of FSEP we compared participant performance in the pre- and post-education assessments. To explore enduring impact of FSEP we compared pre-education assessment scores for first time and repeat participants.

To maintain quality and to evaluate the stability over time of the assessment, we undertake annual psychometric quality assurance (Rasch) analyses of all items. This includes amending or removing poorly functioning items or distractors, and adjusting content as required. A new assessment is prepared each year, within the constraints of a previously published blueprint ^9^.

Based on item quality metrics from the psychometric analysis, we select a range of high-quality anchoring items for each assessment to maintain the reliability and comparability of successive assessment forms. To assess performance consistency of the participant cohorts undertaking the assessment over time, we analysed scores against the anchor set of MCQ items used in each of the 60 MCQ assessments from 2010 to 2018.

To assess consistency of the assessment difficulty over time, we analysed the results of first-time participants across all years. In addition, participant scoring for all craft groups on the assessments, relative to the individual’s previous exposure to the program, was undertaken to measure impact over time.

All comparative statistical analyses were undertaken using Statview (StatView Inc. Nesbit, MS, USA). Parametric data were compared using Student’s t-test. Statistical significance was accorded where p<0.05.

Approval from RANZCOG was granted to use the de-identified assessment results from the FSEP Database, for the development of this publication.

## Results

Between 2004 – 2018, 25,848 clinicians undertook FSEP. The results from 64,430 assessments (100%) were available for inclusion in this analysis. From 2004 to 2006, 3770 clinicians participated in FSEP, undertaking both the pre- and post-education assessments. Of these, 3,506 undertook the education and assessments once, and 274 did so twice.

The immediate impact of the education program on knowledge gain was assessed by comparing participant scoring from their pre-education assessment to their post-education score ( Table 1). On average, a 51% improvement in raw score performance was seen following the education program ( Table 1). The greatest improvement in raw score performance was noted among student midwives, who had a 64% improvement on average, but also the lowest pre-education assessment average scores (8.16 ± SD 2.13). In comparison, O&G registrars showed the least improvement in post-education assessment raw score (33%) but had the highest pre-education scores (12.43 ± SD 2.18; Table 1).

**Table 1.** Combined pre and post assessment scores 2004–2006.

Pre- and post-education assessment scores 2004–2006 combined								
Designation	Pre-test	stdev.s	raw score	Post-test	stdev.s	raw score	Increase	#
**GP / GP Obstetrician**	10.17	2.48	50.8%	15.78	2.62	78.9%	55%	224
**Midwives**	9.24	2.57	46.2%	14.00	2.73	70.0%	52%	2967
**Obstetricians**	11.48	2.63	57.4%	15.69	2.66	78.4%	37%	247
**Other**	8.47	2.65	42.3%	13.75	3.11	68.7%	62%	89
**Registrar**	12.43	2.18	62.1%	16.57	2.01	82.8%	33%	118
**Resident**	9.77	2.60	48.9%	15.41	2.66	77.0%	58%	157
**Student midwife**	8.16	2.13	40.8%	13.35	2.70	66.8%	64%	232

**Figure 1.**
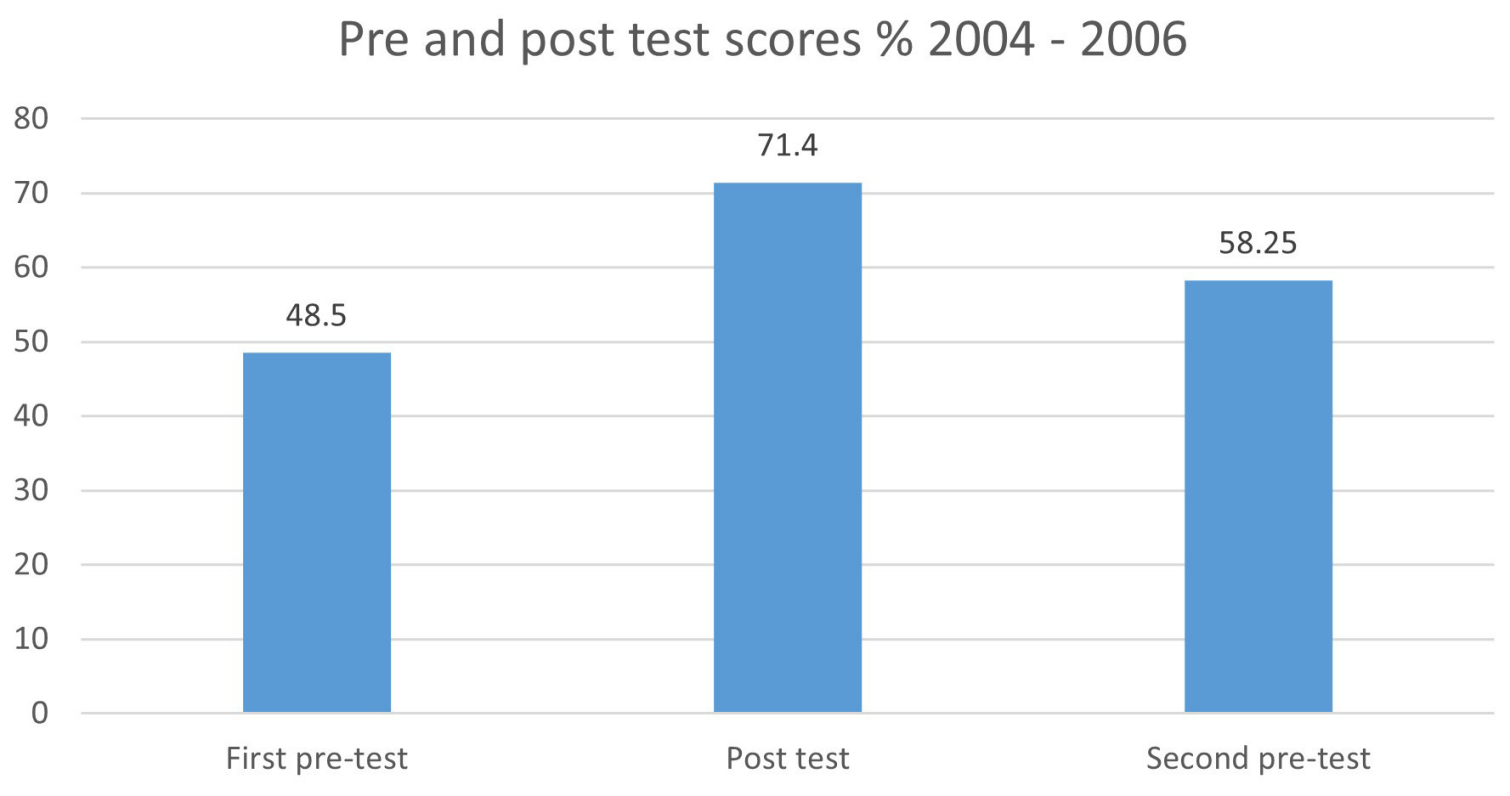
Pre and post test scores % 2004–2006.

To assess for medium-term scoring improvement, the results of the 264 participants who attended the program twice during this period (2004–2006) were analysed. Participants’ first pre-education assessment scores were compared with their second pre-education scores, 12 to 18 months later ( Figure 1). This confirmed that knowledge retention was occurring in the medium-term, with improved pre-education assessment scores noted the second time each of these cohorts participated, at 48.5% (SD 2.50) and 58.25% (SD 2.77) respectively. Although participant numbers for the second pre-assessment are modest, this suggests 40% of the original 50% improvement in scoring is maintained over a 12–18-month period.

**Figure 2.**
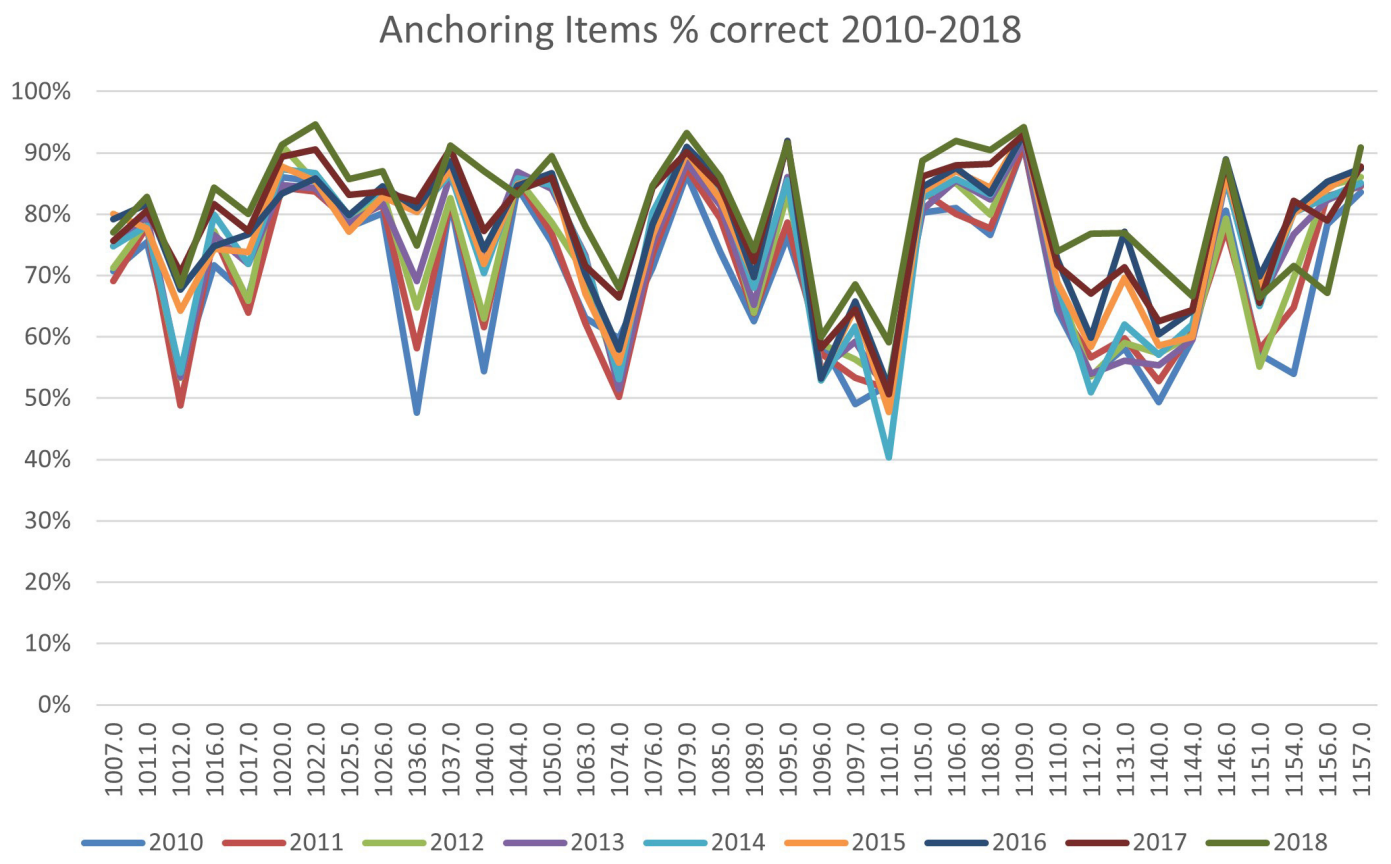
Anchoring Items % correct 2010–2018.

The facility index (percentage correct) for the 38 anchoring items used in the 60 item MCQ assessment each year between 2010 and 2018, are illustrated in Figure 2. With minor exceptions, analysis of the anchoring items used in the assessments between 2010 and 2018 shows most items performing consistently in terms of their relative and absolute difficulties between years. This suggests the proportion of professions and skill level in the cohorts tested, are broadly stable from year to year, confirming the overall stability of the assessment tool itself.

**Figure 3.**
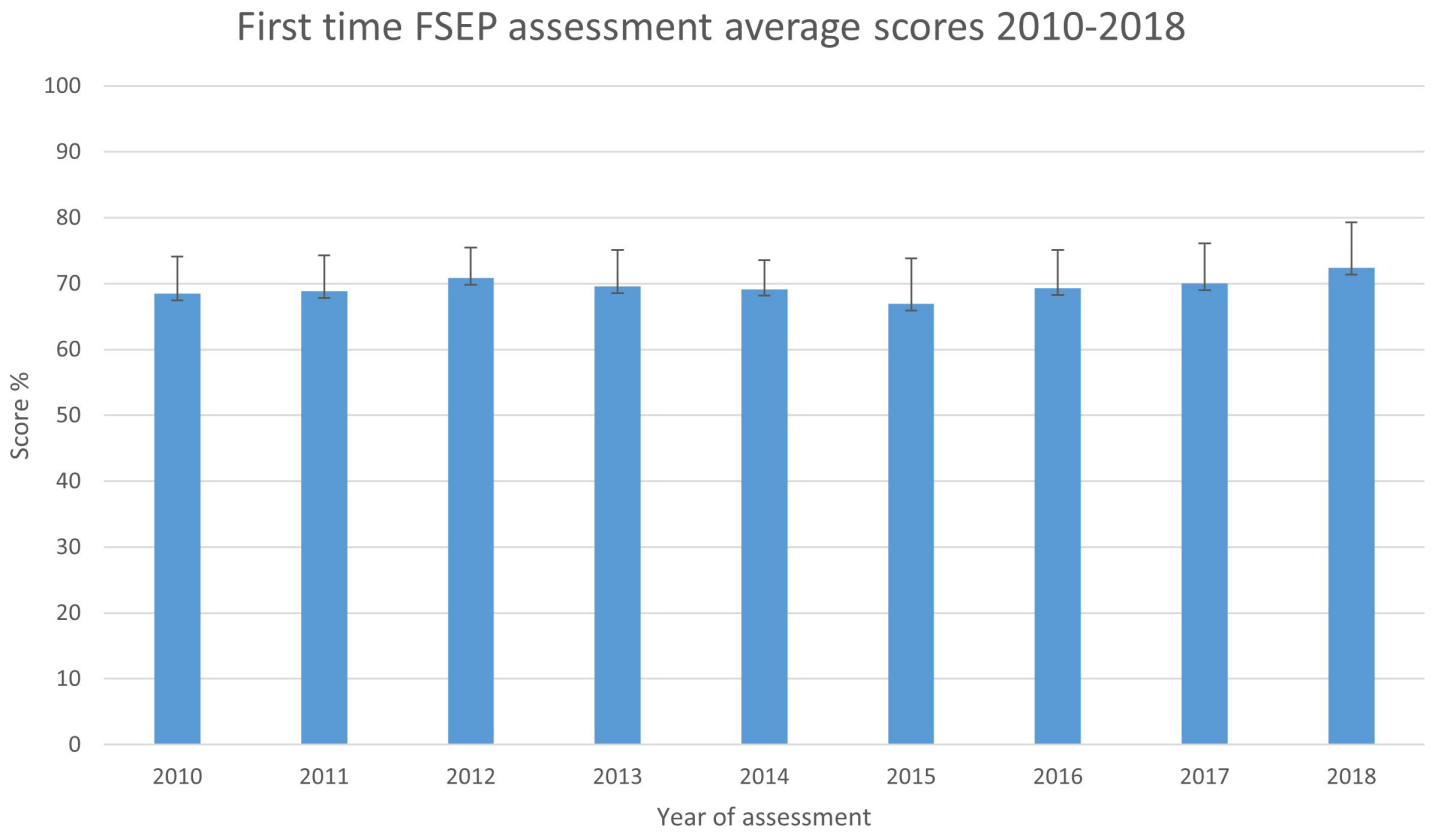
First time FSEP assessment average scores 2010–2018.

On average, 5,500 participants undertook the education program and assessment each year between 2010 and 2018, with the average first time participants raw score stable over the 9 years at 69.50% (SD 1.52; Figure 3). This shows the relative stability of the assessment, in terms of difficulty, over time. Any changes in mean achievement across the immediate and medium timeframes therefore suggest practitioner knowledge gain, rather than an easier assessment.

**Figure 4.**
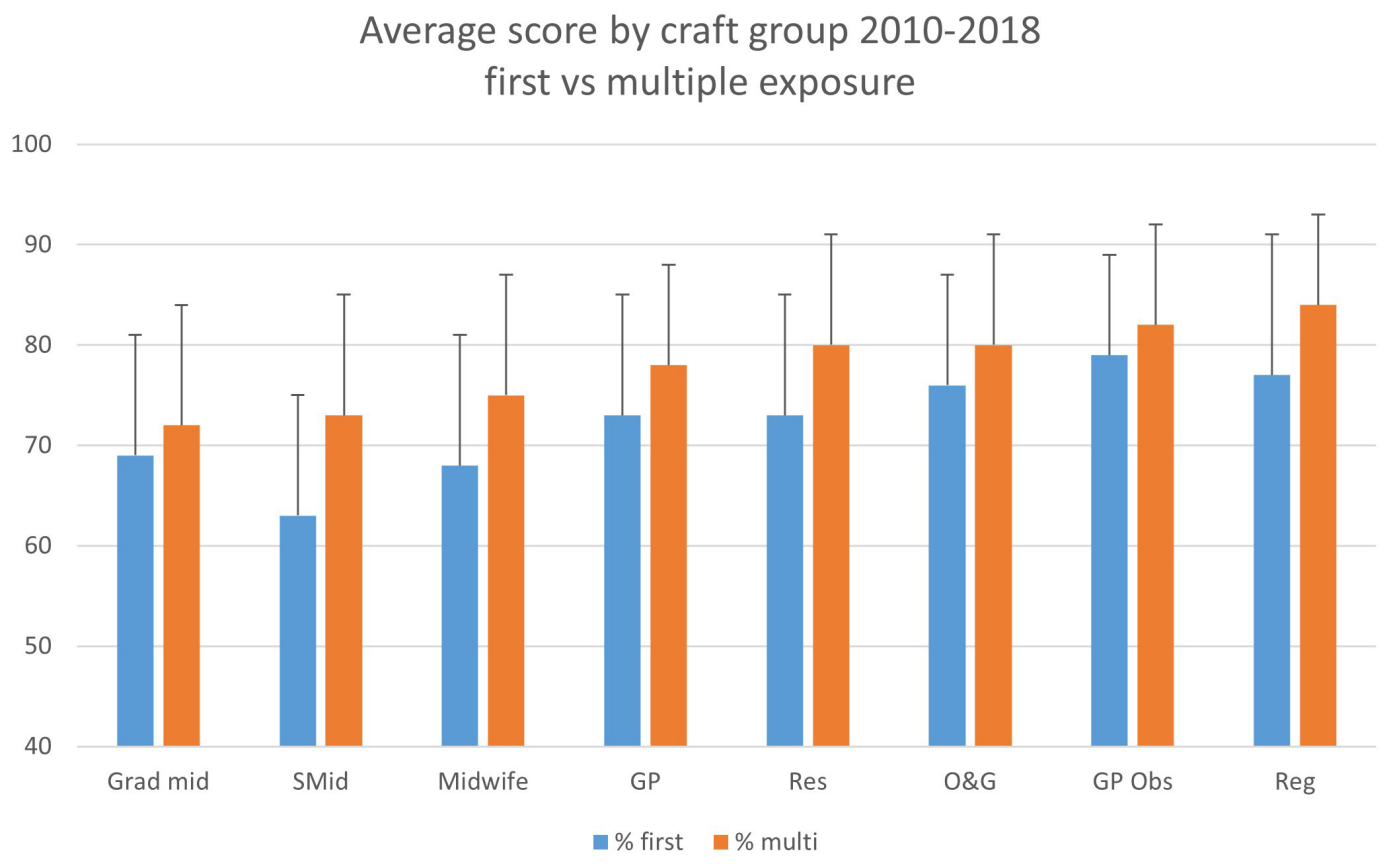
Average score by craft group 2010–2018 first vs multiple exposure.

Participant performance by craft group (2010 – 2018) in the FSEP education and assessment showed improvement in assessment scores with repeated exposure (n = 33,590 assessment results) to the program for all craft groups ( Figure 4). The average scores per craft group are shown in Table 2. The highest scoring cohort each year ( Figure 4) is noted to consistently be the participants with previous exposures to the FSEP program. This confirms an improved performance in the assessment with ongoing exposure to the program.

**Table 2.** Average assessment scores by craft group, first vs multiple attempts.

Average assessment score by craft group, 2010–2018, first vs multiple attempts						
Designation	% first	stdev.s	# first	% multiple	stdev.s	# multiple
**Graduate midwife**	69	12	1021	72	12	239
**GP**	73	12	273	78	10	138
**GP Obstetrician**	79	10	233	82	10	252
**Midwives**	68	13	10887	75	12	10814
**Obstetrician**	76	11	722	80	11	718
**Registrar**	77	14	1017	84	9	605
**Resident**	73	12	1006	80	11	208
**Student midwife**	63	12	285	73	12	154

## Discussion

The development and delivery of the FSEP program was intended to meet an educational gap and improve clinicians’ knowledge and associated cognitive skills in CTG use, interpretation, and management. It was hoped that this might assist in reducing adverse outcomes arising from the incorrect use and/or interpretation of CTGs. Here we have demonstrated a short- and medium-term improvement in clinician scoring in the FSEP assessment, for all craft groups, following education. We also demonstrated continued improvement in CTG knowledge and cognitive skills with repeat exposure to the education and assessment program. These achievements were seen across all craft groups and were not attributable to an easier assessment.

Our work provides a comprehensive addition to the literature, supporting the findings of the Pehrson *et al.* systematic review that indicated that CTG training was associated with improvements in CTG knowledge, interpretive skills, interobserver agreement and clinical competence, though recognising most included studies were of modest quality ^11^. A subsequent systematic review and meta-analysis by Kelly *et al.* (2021) ^12^, also suggested that while there were improvements in CTG knowledge following education; evidence for the optimal content and method of delivery of training was limited. Both reviews noted that further evaluation of training, as well as the impact on outcomes, was needed.

There have long been calls for CTG education and training to be more physiologically based ^13– 15^, as opposed to the long-standing pattern recognition approach, which typically lacks appreciation of the underlying pathophysiology. Indeed, the positive impact of fetal physiology training on CTG interpretation and management has recently been shown in two studies ^16, 17^. While these studies, with a similar physiological approach to their education as our program, showed improvements in interpretation accuracy, assessment scoring and homogenisation of management decisions, participant numbers were modest.

Some have made the case for mandatory CTG training and competency assessment ^18– 20^. It has also been argued that any competency/credentialing assessment tool must have established validity and reliability and be supported by ongoing quality assurance ^21^; integral components in the FSEP program explored here. 

The strength of this study is that our investigation of the FSEP program on fetomaternal physiology, CTG interpretation and knowledge gain is underpinned by an assessment tool for which validity and statistical reliability have been established ^9^. With ongoing annual quality assurance ensuring test reliability and accurate data collection, the ability to track test impact on knowledge retention over time and across multiple course attendances is possible. In addition, the size of this study, consisting of 64,430 test results from 25,848 clinicians, is the largest yet performed. The longitudinal data, combined with the varying skill-mix of participating craft groups, ensures a broad and robust analysis of the impact of FSEP on clinician knowledge gain.

Limitations do exist however, and we acknowledge the possibility that repeat exposure to some questions could lead to a small memorisation/familiarity effect in the ‘repeat exposure’ cohort. We would expect any impact to be minor given that the exposure is usually over a year apart.

We also note that contrasting outcomes from alternative modelling frameworks may provide additional measures of performance improvements and a means to evaluate the robustness of the findings reported here.

While suggestions of improved fetal outcomes have been made following FSEP education training and assessment ^21, 22^, causation remains unproven. Additionally, ongoing refinements, particularly in the early years of the program have limited the ability to monitor longitudinal benefits with repeated exposures over a period longer than 9 years. Such evaluation in the future could assist with understanding the frequency of program attendance required to achieve and maintain the knowledge gains evident in this study. This would assist with informing future health policy credentialing recommendations.

## Conclusion

We have shown that the FSEP education program led to improved clinician knowledge and cognitive skills in the short and medium term. This is apparent for all craft groups. That the FSEP assessment works effectively for all craft groups is important, because maintaining multiple reliable and discriminatory assessments would be both difficult and time consuming. Additionally, given the multidisciplinary nature of maternity care in Australia, a single high quality education program and robust assessment tool, might assist with consistent and standardised approaches to interpretation and care. This should help to optimise equality in care for women across a range of maternity service levels and providers.

Importantly, clinician performance on the assessment continues to improve with ongoing exposure to the education program. That improved scoring by clinicians in the FSEP assessment is associated with increased educational exposure is both intuitive and desirable. That this translates to improved clinical outcomes is uncertain and warrants further investigation.

## Data Availability

Figshare: Pre and post education assessment results, 2004–2018, from the RANZCOG Fetal Surveillance (CTG) Education Program, https://doi.org/10.6084/m9.figshare.22707370.v1 ^23^. This project contains the following underlying data: combined 04,05,06 data.xls Assessment ANCHORING item numbers 2010 TO 2018 data check 5.xlsx Figshare: Pre and post education assessment results, 2004–2018, from the RANZCOG Fetal Surveillance (CTG) Education Program, https://doi.org/10.6084/m9.figshare.22707370.v1 ^23^. This project contains the following extended data: Statistical Report PDFs Data are available under the terms of the Creative Commons Attribution 4.0 International license (CC-BY 4.0).
