# Supplementary material for: Immediate and longer-term impacts of fetal surveillance education on workforce knowledge and cognitive skills
Source: MedEdPublish (2016). 2023 Jul 19;13:38. [Version 1] doi: 10.12688/mep.19656.1 (PMC11109546; doi:10.12688/mep.19656.1)
Supplement: Peer review history

# Reviewer report: Reviewer response for version 1

**DOI:** 10.21956/mep.21056.r36723

**Published:** 2024-07-02

**Authors:** Malin Holzmann

**Affiliations:** 1 Dept Women's and Children's Health, Karolinska Institutet, Stockholm, Stockholm County, Sweden; 2 Medical Unit Pregnancy and Delivery Care, Karolinska University Hospital, Stockholm, Sweden

This is a thoroughly designed and well-performed study regarding the impact of a nationwide education program on knowledge skills both among different professions and over time.

 In summary, an education program on intrapartum fetal surveillance was launched in 2004 and besides education, containing an MCQ test. From 2004 to 2006, pre- and post-education tests were performed, and from 2007, only post-education tests. The study results consist of 64,430 test results from 25,848 clinicians.

 In the comparison before and after the education, there was a 51% improvement on average. The largest improvement was seen among student midwives, who also had the lowest pre-education scores. The least improvement was seen among O&G registrars who on the other hand had the highest pre-education test results.

 There was a significant knowledge retention and medium-term improvement with a higher pre-education test score prior to the second education.

 When comparing the post education test results between the years 2010 and 2018, there was a significant improvement with repeated exposure to the program for all craft groups.

 Language and grammar are satisfying, the design of the study is impressive and appropriate.

 Methods and analyses are clearly described, the interpretation of data is sound, and conclusions are supported by the results.

 One strength of the study is the large sample size. The thorough way of validating the stability of the test over time, and the performance consistency of the study cohort is clearly described. The researchers have also assessed consistency of the test difficulty over time which they show is stable and reliable.

 Interpretation of data is sound, all conclusions are supported by the results, and the authors identify proper limitations of the study.

 Links to the source data is provided in the manuscript. I do not find a single critic or suggestion of improvement of the manuscript.

**Minor comments.**

 1) There are a few weaknesses of which some are addressed by the authors. Besides their mentioned limitation regarding a possible memorization/familiarity effect, another reflection might be worth having in mind while reading the results.

 2) There is no comparison group who did not undertake any education at all during the study years. All clinicians probably develop during regular work as they gradually gain clinical experience. As the learning curve often is steepest among junior staff, this might have influenced the study results for student midwives for example.

 3) However, in my opinion, the study is relevant for all countries that have national education programs in fetal surveillance for labor staff.

Have any limitations of the research been acknowledged?

Yes

Is the work clearly and accurately presented and does it cite the current literature?

Yes

If applicable, is the statistical analysis and its interpretation appropriate?

Yes

Are all the source data underlying the results available to ensure full reproducibility?

Yes

Is the study design appropriate and is the work technically sound?

Yes

Are the conclusions drawn adequately supported by the results?

Yes

Are sufficient details of methods and analysis provided to allow replication by others?

Yes

Reviewer Expertise:

fetal surveillance, electronic fetal monitoring, biochemical fetal monitoring, computerized cardiotocography

I confirm that I have read this submission and believe that I have an appropriate level of expertise to confirm that it is of an acceptable scientific standard.

# Reviewer report: Reviewer response for version 1

**DOI:** 10.21956/mep.21056.r35511

**Published:** 2024-05-25

**Authors:** Sara Creighton

**Affiliations:** 1 Department of Pediatrics, Division of Pediatric/ Fetal Cardiology, Froedtert & the Medical College of Wisconsin (Ringgold ID: 37353), Milwaukee, Wisconsin, USA

This study looks at the effectiveness of the FSEP program to educate clinicians about fetal surveillance in Australia and New Zealand which includes a course and then pre-/post- assessments. The introduction adequately describes the course itself but would expand on the assessment tool that the study is discussing as this was a retrospective review and the assessment tool was not an intervention. In addition it may be helpful to discuss the types of questions involved in the assessment of the participant (multiple choice vs narrative). 

 The methods of the study adequately describe the differences over time in the assessment tool. I found the description of the statistical analysis less clear especially when looking at the results. The methods discuss calculation of p-values for significance but I only see standard deviations reported in the results. There is no listed statistical significance to the data and may be beneficial to give pre vs post test p-values and maybe even compare "student" type. 

  The Results section shows the differences in the pre/post test as well as the post-test only over time but I think these could be re-organized to show P-values (would also put the # of participants on the left). As for Figure1 I would want to know what the standard deviations were for the scores as well as the second post-test score. I'm not sure if there is a better way to show the Anchoring data in Figure 2 (including better labelling of the axes). Figure 4 is a good graph to show scores over time but would recommend showing P-values showing if there was any statistical differences. 

 The discussions had great data cited to show the benefit of the FSEP and previous data. I do think that there could be some better discussion if you could utilize statistical significance for the data.

 There is a piece of the discussion stating that the assessment tool is validated and statistically reliable which may need to be also stated in the introduction. I feel like the hypothesis to see if there is improved knowledge over time could be better stated in the conclusion

Have any limitations of the research been acknowledged?

Yes

Is the work clearly and accurately presented and does it cite the current literature?

Yes

If applicable, is the statistical analysis and its interpretation appropriate?

Partly

Are all the source data underlying the results available to ensure full reproducibility?

Yes

Is the study design appropriate and is the work technically sound?

Partly

Are the conclusions drawn adequately supported by the results?

Partly

Are sufficient details of methods and analysis provided to allow replication by others?

Yes

Reviewer Expertise:

Fetal Cardiology, Risk assessment of fetal cardiac patients, Genetic etiology of cardiac disease

I confirm that I have read this submission and believe that I have an appropriate level of expertise to confirm that it is of an acceptable scientific standard, however I have significant reservations, as outlined above.

# Reviewer report: Reviewer response for version 1

**DOI:** 10.21956/mep.21056.r35657

**Published:** 2024-05-25

**Authors:** Richard B. Hays

**Affiliations:** 1 College of Medicine and Dentistry, James Cook University, Townsville, Qld, Australia

While this paper reports an interesting CPD intervention that is inter-professional, the methods are relatively weak, weakening any conclusions that can be drawn.

 The finding that knowledge improves from just before to just after an educational intervention is almost guaranteed unless there is a problem with the intervention or assessment.  This means that the intervention may be well-designed and implemented appropriately.  However, it is well known that such knowledge decays quite quickly.  Focusing on repeating the course and repeating knowledge assessments should also be expected to work, but this is all about 'knows' and 'knows how', whereas the more important level is 'does'.  The best way of achieving that is through WBA and care outcomes.  This makes the paper much less innovative and of more marginal value.  Could the authors attempt to link their data with WBA and real patient outcomes?  If not. expanding on limitations should be considered.

Have any limitations of the research been acknowledged?

Partly

Is the work clearly and accurately presented and does it cite the current literature?

Yes

If applicable, is the statistical analysis and its interpretation appropriate?

Yes

Are all the source data underlying the results available to ensure full reproducibility?

Yes

Is the study design appropriate and is the work technically sound?

Partly

Are the conclusions drawn adequately supported by the results?

Partly

Are sufficient details of methods and analysis provided to allow replication by others?

Yes

Reviewer Expertise:

Medical education & rural health

I confirm that I have read this submission and believe that I have an appropriate level of expertise to confirm that it is of an acceptable scientific standard, however I have significant reservations, as outlined above.

# Reviewer report: Reviewer response for version 1

**DOI:** 10.21956/mep.21056.r36722

**Published:** 2024-05-10

**Authors:** Deborah Fox

**Affiliations:** 1 University of Technology Sydney, Broadway, New South Wales, Australia

The RANZCOG Fetal Surveillance Education Program is recognised as a robust training program for Australian obstetricians, midwives, GP Obstetricians and students in the interpretation of CTG data. This study supports its reputation by clearly demonstrating an improvement in clinician interpretation, based on the results from a large pre and post assessment data set collected between 2004 and 2018. Quality of interpretation of CTG data is one component of a complex group of factors contributing to the safety of women and their unborn babies in childbearing and this is an important addition to the literature on fetal surveillance education.

Have any limitations of the research been acknowledged?

Yes

Is the work clearly and accurately presented and does it cite the current literature?

Yes

If applicable, is the statistical analysis and its interpretation appropriate?

I cannot comment. A qualified statistician is required.

Are all the source data underlying the results available to ensure full reproducibility?

Yes

Is the study design appropriate and is the work technically sound?

Yes

Are the conclusions drawn adequately supported by the results?

Yes

Are sufficient details of methods and analysis provided to allow replication by others?

Yes

Reviewer Expertise:

Midwifery, intrapartum care, continuous electronic fetal monitoring.

I confirm that I have read this submission and believe that I have an appropriate level of expertise to confirm that it is of an acceptable scientific standard.

# Reviewer report: Reviewer response for version 1

**DOI:** 10.21956/mep.21056.r35663

**Published:** 2024-03-01

**Authors:** Sharon Dore

**Affiliations:** 1 McMaster University, Hamilton, Ontario, Canada

This article reviewed the value of a national full day cardiotocography (fetal health surveillance) education program in Australia New Zealand. Results of a large sample of pre and post MCQ demonstrated knowledge and application of knowledge over time of all disciplines of care providers. (Obstetricians, residents, trainees, General Practitioners, midwives and student midwives). Outcomes showed knowledge retention was 18 months for all groups. Repeating program during the analysis period (2004-2018 ) improved performance.

 This study adds to the evidence that formal education programs, based on physiology, application of knowledge and decision making, improve fetal heart rate interpretation. It aligns with the current Canadian guideline recommendation of completing the Canadian national education program initially and repeating the program every 2 years.  Emphasis on interprofessional cardiotocography education will lead to improved care and clinical outcomes and should be emphasized in all learner programs and  hospital accreditation and quality reviews.

 Reference:

 [1]

Have any limitations of the research been acknowledged?

Yes

Is the work clearly and accurately presented and does it cite the current literature?

Yes

If applicable, is the statistical analysis and its interpretation appropriate?

Yes

Are all the source data underlying the results available to ensure full reproducibility?

Yes

Is the study design appropriate and is the work technically sound?

Yes

Are the conclusions drawn adequately supported by the results?

Yes

Are sufficient details of methods and analysis provided to allow replication by others?

Yes

Reviewer Expertise:

fetal health surveillance, maternal mortality, education

I confirm that I have read this submission and believe that I have an appropriate level of expertise to confirm that it is of an acceptable scientific standard.

## References

[1] : No. 396-Fetal Health Surveillance: Intrapartum Consensus Guideline. *J Obstet Gynaecol Can* .2020;42(3) : 10.1016/j.jogc.2019.05.007 316-348.e9 10.1016/j.jogc.2019.05.007 32178781
